# A high-brightness large-diameter graphene coated point cathode field emission electron source

**DOI:** 10.1038/s41467-018-03721-y

**Published:** 2018-03-29

**Authors:** Xiuyuan Shao, Avinash Srinivasan, Wei Kean Ang, Anjam Khursheed

**Affiliations:** 1 Department of Electrical and Computer Engineering, National University of Singapore, 4 Engineering Drive 3, Singapore, 117583 Singapore; 2 Engineering Science Programme, National University of Singapore, 9 Engineering Drive 1, Singapore, 117575 Singapore

## Abstract

There have been several long-standing problems of cold field emission sources for electron microscopy and lithography that have prevented their widespread use, such as their inherent ultrahigh vacuum condition requirement (<10^–9^ torr), relatively poor current stability and rapid emission decay. This paper presents a cold field emission electron source which overcomes these problems based upon using a graphene-coated nickel point cathode. Preliminary experiments demonstrate that it provides stable emission for relatively large tip diameters (micron sizes), can operate in high vacuum conditions (>10^−8^ torr) and has an ultralow work function value of 1.10 ± 0.07 eV. It has an estimated reduced brightness value of 1.46 × 10^9^ A m^−2^ sr^−1^ V^−1^ for cathode tip-radius of 170 nm and the measured energy spread ranges from 0.246 eV to 0.420 eV for a tip radii range of 260 nm to 500 nm, which is comparable to state-of-the-art conventional cold field emission sources.

## Abstract (web summary)

Traditional cold field emission electron sources rely on single crystal tungsten sharpened wires. Here, the authors use graphene-coated nickel point cathodes to devise alternative cold field emission electron sources for electron microscopy and lithography applications.

## Introduction

Within the wide variety of electron sources used for electron microscopy and electron beam lithography, cold field emission electron sources have the highest brightness, coherence and lowest energy spread, making them the best choice for high-resolution applications^1–4^. The most common type of cold field emission electron source in high-resolution microscopy usually utilizes a single crystal (310)-oriented tungsten sharpened wire as the cathode, whose tip radius is in the 100–200 nm range with a work function (*ϕ*) of 4.4 eV^5^. Typically, electron emission from the cathode occurs when the field strength at the cathode tip exceeds values around 4 V nm^−1^, allowing electrons to escape from the cathode surface by quantum tunneling^5^.

Despite their desirable electron optics properties, cold field electron emission sources suffer from some well-known practical difficulties that have prevented their widespread use. Two such long-standing technological challenges include, firstly, their requirement of ultrahigh vacuum (UHV) conditions (<10^−9^ torr for W(310)) and, secondly, their inherent current instabilities, both in their long-term and short-term emission^5^. As a result, the stable and reliable Schottky electron beam source, which involves heating the cathode to 1800 K, is the most widely used type of electron source for high-resolution electron microscopy applications, despite it having significantly lower brightness, coherence and higher energy spreads than cold field emission sources^6,7^. Another important limitation of conventional cold field emission electron sources is that their emission current decays significantly (about 70–90% of the initial value) within the first hour of usage, subsequently requiring cathode flashing (Joule heating at high temperatures) at regular intervals, as short as a few hours, to reach the original emission current levels^5^.

Although a variety of other possible types of cold field emission cathodes have been reported in the past decades, such as a tungsten nanowire^8^, a carbon nanotube^2,9,10^, a carbon nanotip^4,7^ and a LaB_6_ nanowire^3^, their practical difficulties are in general even more severe than for the conventional single crystal sharpened tungsten wire cathode source: the vacuum pressure requirement needs to be typically lower than 10^−10^ torr for nanowire-based field emitters, otherwise the emission currents will suffer from large fluctuation and rapid emission decay.

One material, reported to have good field emission properties, but not yet used in cold field emission sources for electron microscopy/lithography applications, is graphene. Graphene, a single layer of carbon atoms arranged in hexagonal lattices, is well known for having a wide range of promising applications owing to its excellent thermal, mechanical and electrical properties^11–13^. Cold field emission from graphene has been reported in the context of creating micro-fabricated nanometer-scale sharp protrusions to localize and enhance an applied electric field, either by transferring graphene sheets onto metal/semiconductor nanotips^14–16^, graphene coating of Ni/Co nanotips^17^, or by forming vertically aligned graphene films^18,19^. However, none of these methods are suitable for electron microscopy/lithography applications since they inevitably create multiple emission sites on the cathode plane. Conventional electron microscopy/lithography columns require a single cathode emission site, which then produces a single nanometer size virtual source point at the gun exit. Conventional single-tip sharpened wire cathodes not only produce single-point virtual sources (typically one to two orders of magnitude smaller than the tip radius (100–200 nm)), but also have relatively large supporting wire dimensions (in the millimeter range), providing mechanical and thermal stability for the field emission. The multiple micro-fabricated emitters involving graphene sheets or graphene coating where graphene field emission has been demonstrated so far requires much more development before it can be applied to the subject of electron beam microscopy and lithography.

So far, the only single-tip emitters involving graphene consist either of overlaying an ultrathin (~1 nm) graphene flake on to a blunted tungsten probe^20^, or a graphene ring structure^21^. In the case of the loosely hanging ultrathin and freestanding graphene flake, it cannot be used for practical applications, since the graphene flake can easily be damaged or detached from the supporting tungsten probe by back bombardment of high-energy positive ions. In the case of the graphene ring cathode, it requires the use of a non-conventional electron beam column, one which forms a ring beam focus at the specimen plane, instead of the normal single-point focus.

Here we report a few-layer graphene-coated nickel (Graphene-Ni) wire cathode in the sub-micron to micron size that can operate in high vacuum (HV) conditions (>10^−8^ torr) with high current density. Stable electron emission has been experimentally obtained from a cold field emission gun with a single-tip cathode diameter in the micron range and in HV conditions. Successful cold field emission from single-tip emitters has up to now only been possible in UHV conditions with sub-micron tip radii, typically in the 0.1 to 0.2 μm range. The experiments reported here were carried out on a few-layer graphene-coated Ni wire cathode design, and demonstrate it to have an ultralow work function (1.10 ± 0.07 eV), requiring around an order of magnitude lower applied electric field strengths (~0.5 V nm^−1^) than comparable conventional single-tip cathodes. This significantly lower electric field strength requirement makes it possible to have field emission from larger diameter tips (in the micron range), and also reduces the kinetic energies of back-bombarding positive ions. This leads to better current stability and less damage to the cathode tip, and also makes it feasible to operate the gun at less stringent vacuum conditions. Based upon experimentally measured angular intensity results, the Graphene-Ni single-tip cathode is predicted to have a higher reduced brightness than comparable conventional cold field emission electron sources, and there is no obvious need for regular thermal flashing of the cathode. These advantages come from its ultralow work function and relatively low applied electric field strength at the tip apex. The single-tip Graphene-Ni wire cathode source as presented here appears to have overcome many of the practical difficulties which have so far hindered the widespread use of cold field emission sources for electron/microscopy applications.

## Results

### Fabrication of the Graphene-Ni electron point source

**Fig. 1:**
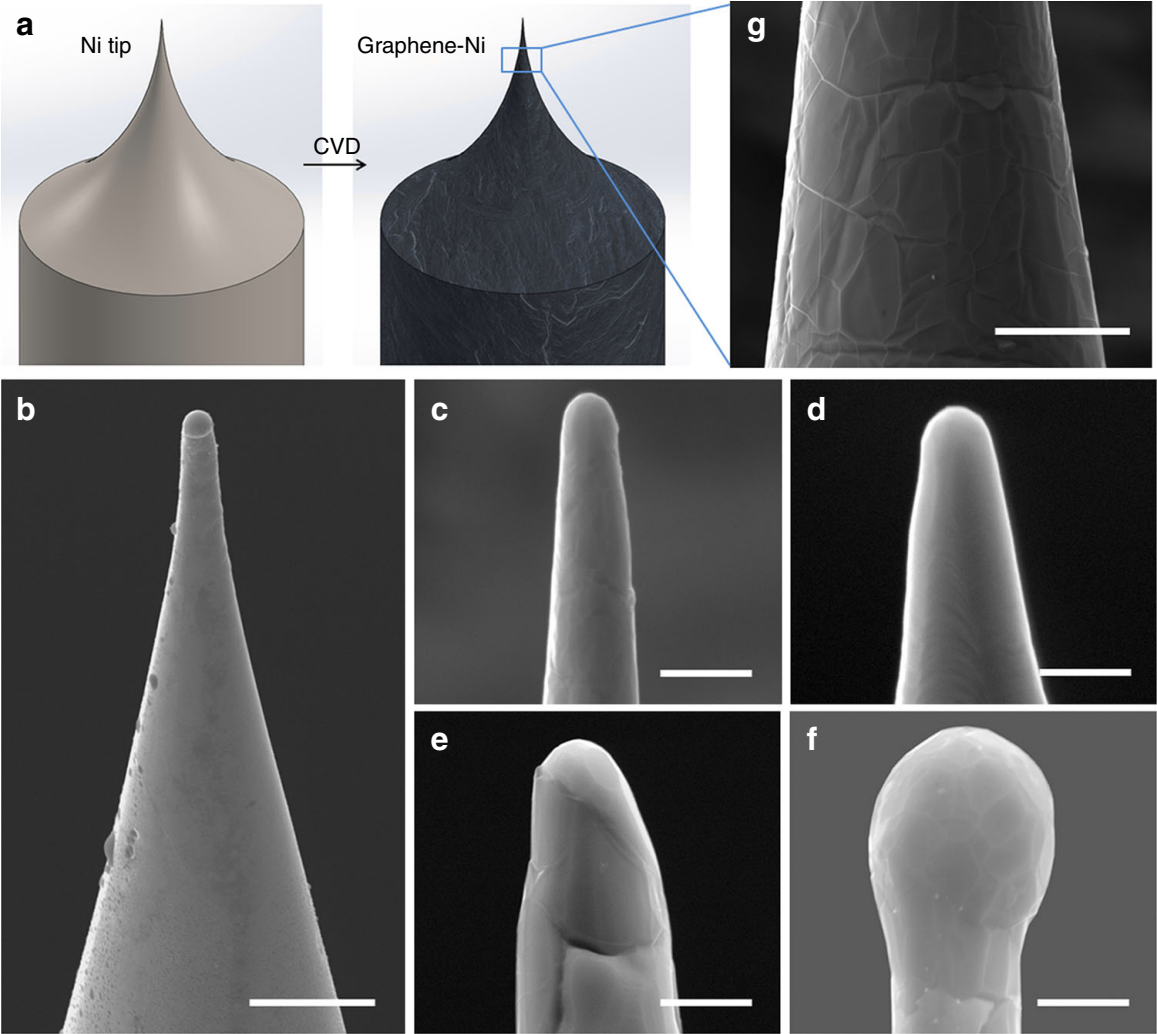
SEM characterization of the Graphene-Ni field emitter. **a** Illustration of the fabrication of a graphene-coated point cathode. **b** SEM images of as-etched Ni tip from electrochemical etching at low magnification showing supporting wire (scale bar, 5 µm), and SEM images of graphene-coated point cathodes of different tip radii: **c** 210 nm, **d** 300 nm, **e** 480 nm and **f** 800 nm. Scale bar (**c**,** d**,** e**,** f**), 1 µm. **g** SEM image of the lateral surface of the emitter. Scale bar, 5 µm

A two-step process (Fig. 1a) was used for the fabrication of Graphene-Ni point cathodes reported in this work. Ni wires of 1 mm diameter were electrochemically etched to obtain a sharp Ni tip with tip radii ranging from 170 nm to 800 nm (Fig. 1b). These were then used as catalysts and templates for the growth of few-layer graphene via chemical vapor deposition (CVD) process at a moderate temperature (<900 °C) to avoid any morphological change to the Ni tip (Supplementary Fig. 1). Detail of this growth process is described in the Methods section of this paper. It is worth mentioning that Ni is not only conducive for the growth of uniform layers of graphene over large areas with high crystallinity^22^, but it also significantly reduces the work function of graphene^23,24^. This lowering of work function is discussed in detail in later parts of this paper. Typical scanning electron microscope (SEM) images of the graphene-coated Ni tips (of four different tip radii) as depicted in Fig. 1c–f show that the electrochemically etched, smooth surface of the Ni tip apex is overlaid with a continuous, homogeneous and smooth graphene film, and no morphological fluctuations are observed. Due to the ultrathin nature of the few-layer graphene, the grain boundaries of the underlying polycrystalline Ni, formed by high temperature annealing during the CVD process, are visible in Fig. 1g. Wrinkles are observed only on the lateral surface of Ni where graphene was grown, which was possibly induced by thermal stress around step edges and defect lines^25^.

**Fig. 2:**
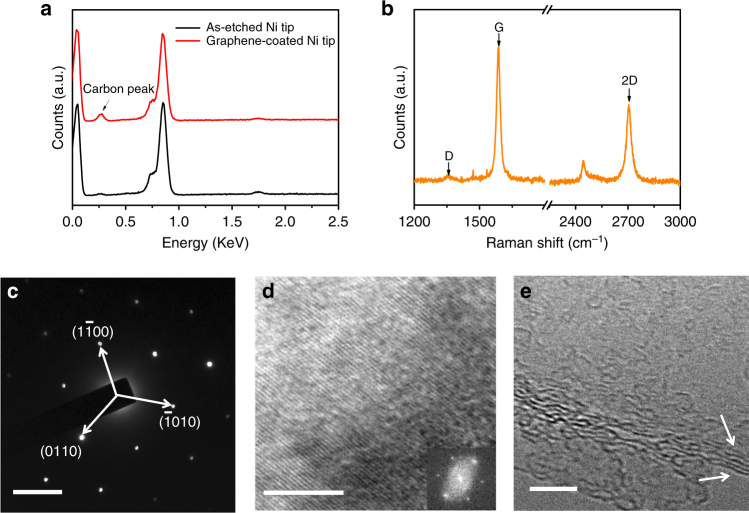
Characterization of the grown graphene. **a** EDS spectra of the Ni tip before (black) and after (red) graphene coating. **b** Typical Raman spectra obtained from the surface of Ni tip coated with graphene with a 532 nm excitation laser. **c** Hexagonal electron diffraction pattern of graphene surface. Scale bar, 5 nm^−1^. **d** High-resolution TEM image of the graphene surface showing the lattice fringes, and inset is the corresponding FFT pattern from the TEM image. **e** HRTEM image on the edge of the graphene flake. Scale bar, 5 nm (**d**, **e**)

To confirm that the Ni cathode wire is covered with graphene after the CVD process, energy-dispersive X-ray spectroscopy (EDS) was employed to examine the chemical composition of the grown film. Figure 2a shows the EDS spectra of the Ni cathode wire, before (black line) and after (red line) the growth of a few-layer graphene. Presence of a carbon peak after the CVD coating process clearly suggests the presence of carbon-based material. Raman spectroscopy was then utilized to determine the type of carbon that is present, e.g., graphene, graphite or amorphous carbon. Figure 2b shows the Raman spectrum that was acquired on the tip surface of the CVD-coated wire cathode. Three characteristic peaks are clearly observed: a D peak at about 1360 cm^−1^, a G peak at about 1586 cm^−1^ and a 2D peak at about 2705 cm^−1^. The position and shape of the G peak confirms the formation of sp^2^ phase carbon and provides further evidence of the presence of graphene^26^. In addition to the relatively high G peak, the D peak which shows the presence of sp^3^ carbon atoms or defects is close to the background level, indicating insignificant defects in the as-grown graphene layers and the film exhibits high graphitic crystallinity^26^. The intensity ratio between 2D and G (*I*_G_/*I*_2D_) peak provides a good measure of the number of graphene layers, and this ratio was around 1.77, a value indicative of a few-layer graphene structure^27^.

A graphene flake was extracted from the surface of the graphene-coated emitter. Selected area electron diffraction (SAED) and high-resolution transmission electron microscopy (HRTEM) were used to further investigate the crystallinity, lattice structure and the thickness of the graphene flake. The SAED pattern (Fig. 2c) of the graphene flake reveals a hexagonal pattern, which signifies its single-crystalline nature, with an in-plane lattice constant of 2.61 ± 0.09 Å (compared to 2.46 Å for graphite^28^). Different regions of the graphene flake were examined and the results consistently indicated the high crystallinity of the deposited graphene film (Supplementary Fig. 2). A high-resolution TEM image is shown in Fig. 2d, with its corresponding fast Fourier transform (FFT) pattern in the inset. The lattice fringes along the in-plane direction of the graphene layer can be clearly observed and its crystallinity is confirmed. The few-layer graphene features are observed on the flake edges from the HRTEM image, as shown in Fig. 2e, which is in agreement with the data obtained using Raman spectroscopy. The interlayer spacing was estimated to be 3.50 ± 0.20 Å.

### Field emission characteristics

**Fig. 3:**
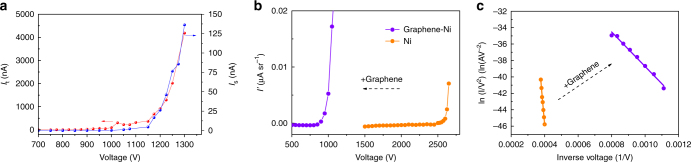
Field electron emission characteristics. **a** The dependence of the total emission current (red) and probe current (blue) on extraction voltage for a tip that has a radius of 400 nm. **b** The angular current density-voltage trace from the Ni tip with a tip radius of 700 nm before coating with graphene (orange), and after coating with graphene (violet). **c** The F-N plot with and without the graphene coating

An experimental cold field emission electron gun setup (Supplementary Fig. 3) was used to obtain the field emission characteristics of the Graphene-Ni point cathodes. The emitter tip was positioned about 0.5 mm away from a grounded anode hole plate having a radius of 0.5 mm, and was aligned to a grounded hole aperture (radius of 0.5 mm) placed in front of a Faraday Cup. The distance of the aperture was adjusted to 16 mm, restricting the semi-angle entry to 30 mrad, corresponding to a collection solid angle of 3 × 10^−3^ sr. The test setup was subsequently placed in a HV (4 × 10^−8^ torr) chamber for field emission tests. A negative voltage *V*_c_ was applied to the cathode (while the rest of the setup was grounded) and the current leaving the anode plate, referred to here as total current (*I*_t_), and the current reaching the Faraday Cup, referred to here as the sample current (*I*_s_), were recorded. The measured (*I*_t_ − *V*_c_) (red) and (*I*_s_ −  *V*_c_) (blue) emission characteristics for a cathode of tip radius of 400 nm are plotted in Fig. 3a. It can be observed that the field emission current increases exponentially as the cathode becomes more negative, following a typical Fowler–Nordheim (F-N) curve^29^. The F-N formula is given as:

1 $$I = A\frac{{1.5 \times 10^{ - 6}}}{\phi }F^2{\mathrm{exp}}\left( { - \frac{{6.44 \times 10^9\phi ^{1.5}}}{F}} \right),$$

where *A* has the dimension of area [m^2^], *ϕ* is the work function in [eV] and *F* is the local electric field on the cathode tip, which is given as $F = \beta V_{{\mathrm{ext}}}/d$ with *β* being the field enhancement factor, $V_{{\mathrm{ext}}}$ the extraction voltage, and *d* the anode to cathode tip spacing. The total current (*I*_t_) reaches a maximum value of 4.2 µA, at which point (*I*_s_) was recorded to be 136 nA, corresponding to a maximum normalized angular current density $I_{\mathrm{N}}^\prime$ value of 34.8 µA sr^−1^ kV^−1^, where $I_{\mathrm{N}}^\prime$ is normalized to the cathode potential. This recorded value of $I_{\mathrm{N}}^\prime$ is approximately 1.4 and 8.6 times greater than that reported for corresponding conventional single crystal tips, W(310) tip and W(111) tip^5,6^, respectively.

One of the stand out features of the Graphene-Ni point cathodes reported here is their ultralow work function values. This significant finding was extracted from the experimental results shown in Fig. 3b, c, which compares the *I*–*V*_c_ characteristics and F-N plot for a Ni tip emitter (radius of 700 nm) before (orange) and after (violet) it is coated with graphene. Work function estimates were extracted from the slope of the graph in the F-N plot, the cathode to anode distance (*d*), and field enhancement factor (*β*), using Supplementary Eq. 4. The distance *d* was measured accurately in a SEM, and SEM imaging also provided an estimate of the tip radius, which was approximated to be spherical in shape. The Lorentz-2EM boundary element software^30^ was then used to numerically solve for the potential field distribution around the tip, and derive a simulated value for the field enhancement factor *β* (Supplementary Fig. 4). The work function estimates from the F-N plots using this method were calculated to be 5.80 eV for the bare Ni tip and 1.03 eV for graphene coating Ni tip. The 5.80 eV value for the bare Ni tip agrees within 6% of the one reported previously for bulk Ni by other researchers^23^, confirming that the work function estimates derived from the present experiment are relatively accurate. Further confirmation of the accuracy comes from calculating the work function for the graphene-coated Ni tip by taking the ratio of the two F-N plots shown in Fig. 3c (with and without graphene coating on the same Ni tip), and using the previously reported work function value for bulk Ni, avoiding the need to use values for both *d* and *β*. The work function value for the graphene-coated Ni tip using this second method was calculated to be 1.1 eV, giving better than 7% agreement with the value (1.03 eV) derived directly from the F-N plot using *d* and *β*. The cathode surface area *S*_p_ which contributes to the current collected by the Faraday Cup, was also estimated using simple direct ray tracing simulations (Supplementary Fig. 5), and this information is used to exclude the possibility of emission from wrinkles in the graphene contributing to the sample current (a detailed description can be found in Supplementary Note 1). There are two mechanisms that help explain the significant lowering of the work function: (i) n-type doping of graphene due to chemisorption on Ni, which reduces the work function to the order of 0.5–1.0 eV^23^, and (ii) an increase in the graphene density of states, caused by the enhanced cathode tip electric field, on the order of 0.5–1.5 V nm^−1^, raising the Fermi level^31,32^. The combination of these two effects is most likely responsible for the dramatic reduction of work function value, as measured here, a value of 1.1 eV which, to the best of our knowledge, is the lowest reported value for single-tip pointed cathodes.

### Brightness estimation

**Fig. 4:**
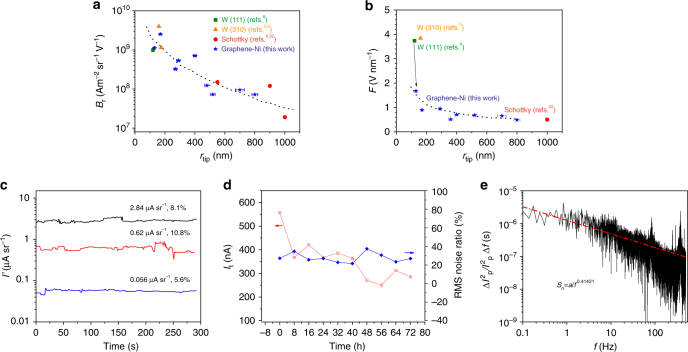
Electron optical characterization of the Graphene-Ni field emitter. **a** Comparison of reduced brightness to other field emission sources. **b** Local electric field strength versus tip radius and the state-of-the-art electron sources. The uncertainty in the *x*-axis of (**a**, **b**) is from the determination of tip size. **c** Short-term current stability curve of the graphene-coated point cathode with a sampling rate of 2 Hz, and the results were obtained in a chamber at a pressure of ~4 × 10^−8^ torr. **d** The averaged total emission current over each period (8 h) versus time for 3 days (red), and the corresponding RMS noise ratio. **e** Power spectrum analysis of the probe current (1.5 nA) with a sampling rate of 1 kHz for a time span of 30 s

For electron microscopy/lithography applications, electron sources of high source reduced brightness *B*_r_ are required, and it can be estimated from the following expression^33^:

2 $$B_{\mathrm{r}} = 1.44\frac{{eI{\prime}}}{{{\mathrm{\pi }} < E_{\mathrm{t}} > }}\left( {\frac{{m_\alpha }}{{r_{{\mathrm{tip}}}}}} \right)^2$$

where *e* is the electron charge, *I*′ is the angular current density, *m*_α_ is the angular magnification, *r*_tip_ is the tip radius and<*E*_t_> is the mean tangential energy. For cold field emission,$\left\langle {E_t} \right\rangle = e\hbar F{\mathrm{/}}\surd \left( {8m\phi } \right)$^33^, where *ħ* is the reduced Planck constant and *m* is the electron mass. Direct ray tracing of electron trajectory paths leaving the cathode surface using the Lorentz-2EM software were carried out to estimate *m*_α_ and *F* and when used together with the measured angular current density *I*, gun brightness *B*_r_ estimates were obtained (Supplementary Note 2). *B*_r_ values for graphene-coated point cathodes versus their tip radii are presented in Fig. 4a. The data in the plot can be broadly grouped into three categories: high brightness tungsten cold field emitters with very small tip radii (<200 nm)^5,6^, Schottky emitters having relatively low brightness with large tip radii (~1000 nm)^34^ and graphene-coated point cathodes having tip dimensions that roughly fall in the middle region. The estimated *B*_r_ value of 2.51 × 10^9^ A m^−2^ sr^−1^ V^−1^ of a graphene-coated cathode of tip radius 170 nm is very close to the highest reported *B*_r_ value of 3.98 × 10^9^ A m^−2^ sr^−1^ V^−1^ for a conventional tungsten cold field W(310) emitter with a tip radius of 160 nm^5^, and is higher than the value of 9.86 × 10^8^ A m^−2^ sr^−1^ V^−1^ for a W(111) emitter with a tip radius of 120 nm^29^. It is worth noting that *B*_r_ values from even larger size tips are still relatively high and comparable to the *B*_r_ value obtained from the state-of-art tungsten field emitters. A summary of the typical operating parameters for the graphene-coated point cathode is listed in Supplementary Table 1. Clearly, the graphene-coated point cathodes exhibit relatively large values of *B*_r_ due primarily to the large value of *I*′ and the substantially small value of <*E*_t_> as a result of the low local electric field strength *F* required to produce electron emission. A high brightness electron beam is affected by statistical Coulomb interactions (Supplementary Note 3), leading to a radial broadening of the virtual source size due to lateral interactions which effectively reduces the source brightness^35^. This effect was calculated for the data of graphene-coated Ni point cathodes (170 nm, 400 nm and 800 nm). The results predict that the brightness will only be lowered by Coulomb interaction effect significantly for the smaller Graphene-Ni cathode tip radius (reduction by around 30% for the 170 nm tip radius), and is not expected to significantly lower the brightness estimates for the larger Graphene-Ni cathode tip radii (Supplementary Table 2).

### Stability of the Graphene-Ni electron point source

The stability of the electron beam is of major concern for focused electron beam applications. Conventional cold field emission electron sources are prone to instability due to the dynamics of residual gas adsorption and ion back bombardment. One of the most important advantages of Graphene-Ni point cathodes over the conventional metal cold field emitters is the chemical inertness of its carbon surface^36^, which is less likely to adsorb residual gas molecules and obviously much more stable. In addition, a lower turn-on electric field is desirable for cold field emitters since it will reduce the kinetic energies up to which the back-bombarding gas-ions are accelerated to when they collide with the cathode surface. The local electric field strength, *F*, required to achieve an angular current density of 5 µA sr^−1^for Graphene-Ni point cathodes of different tip radii are plotted in Fig. 4b. The local electric field strength was estimated by using the enhancement factor derived from simulation (Supplementary Fig. 4b). For comparison, the electric field strength required to obtain the same angular current density from widely used tungsten cold field emitters^5,6^ and Schottky thermal emitters^34^, as reported previously, are plotted on the same graph. It is clear that there is around an order of magnitude reduction in the local electric field strength requirement for the graphene-coated pointed cathodes as compared to the field strength required for tungsten cold field emitters, typically in the range of 0.49–1.67 V nm^−1^, which is comparable to the value of 0.5 V nm^−1^ reported for the Schottky thermal field emitter. These findings help to explain why the graphene-coated point cathode is able to provide stable field emission for micron diameter cathode tips and operate in much less stringent vacuum conditions.

The degree of current instability and damage to the cathode tip not only depends on the chemical inertness of the carbon surface and the kinetic energies of the back-bombardment ions, but also the size of the cathode tip. It is theoretically predicted that the root mean square (RMS) noise ratio (<$\delta I^2$>^1/2^/$\bar I$) varies inversely with the emission surface area, under constant conditions of temperature and pressure^5,37^. Since for the same emission angle, a larger radius tip has a greater area of emission, the relatively large-diameter graphene-coated cathode tips (in the micrometer range), as reported in this paper, are therefore expected to have an order of magnitude lower RMS noise ratio values as compared to conventional tungsten cold field emitters. Figure 4c depicts the typical short-term current stability (~300 s) of a graphene-coated point cathode (tip radius of 360 nm) for sample currents (*I*_s_) of ~0.168 nA, ~1.86 nA and ~8.52 nA, which corresponds to angular current densities of ~0.056 µA sr^−1^, ~0.62 µA sr^−1^ and ~2.84 µA sr^−1^ respectively. Stepwise changes in emission currents were observed and such fluctuations may be attributed to events such as adsorption, desorption or flip-flop of adsorbate molecules at the tip of the emitter^38^. The short-term RMS noise ratios were calculated to be in the range of 5–10%, relatively low for the HV conditions in which they were taken, confirming the better performance expected by theoretical considerations (for larger tip sizes). To further verify the structural robustness of the graphene-coated point cathodes, $I_{\mathrm{t}} - t$ traces were obtained by recording *I*_t_ every 8 h for 3 days as shown in Fig. 4d. The total current was initially observed to be around 560 nA, which decreased to 370 nA in the first 8 h, beyond which a further decay of around 20% was observed over 64 h. This good long-term current stability is much better than for conventional tungsten field emitters whose emission decay can be as high as 70–90% within the first hour of operation, after which periodic flashing is required^5^. In the case of the graphene-coated cathode, there was no obvious need for flashing of the cathode tip. The RMS noise ratios were found to be between 20 and 30% in each 8 h period throughout the 3-day test duration. Repeated field emission tests of the graphene-coated point cathode were carried out in a HV chamber, confirming that the graphene-coated point cathode has highly repeatable field emission characteristics (see Supplementary Fig. 6 and Supplementary Note 4).

The frequency characteristics of the electron emission process was investigated by plotting the normalized spectral density $S_n\left( f \right) = \delta I^2/\bar I^2\Delta f$ of the experimentally detected probe current, as shown in the Fig. 4e. The spectrum was obtained by the FFT of a probe current (1.5 nA) with a sampling rate of 1 kHz for a time span of 30 s. Integration leads to the RMS noise ratio percentage <$\delta I^2$>^1/2^/$\bar I$ = 0.413% or a signal to noise ratio of 242 in the frequency range 0.1–25 Hz. This measured value is lower than that obtained from a conventional tungsten cold field emitter^39^ (~2–5%, *P* ~10^−10^ torr, *T* = 25 °C), and is comparable to that obtained from carbon nanotubes emitters^40^ (~0.2%, *P*~6 × 10^−8^ torr, *T* = 500 °C, *I* = 2.4 nA) and Schottky emitters^41^ (~0.23%, *P*~10^−8^ torr, *T* = 1527 °C, *I* = 30 nA) for the same frequency range.

### Analytically calculated energy spread of the Graphene-Ni electron point source

A preliminary estimate of the energy spread was carried out predicting that the graphene-coated Ni point cathode exhibits comparable energy spread to the conventional W(310) cold field emitter (Supplementary Note 5). Supplementary Fig. 7 shows the room-temperature total energy distribution (TED) for a typical W(310) cold field emitter as well as a graphene-coated Ni tip. From the plots, the intrinsic full-width at half-maximum (FWHM) energy spreads are calculated to be 0.23 and 0.14 eV, respectively. The intrinsic TED of electron emission is only one contributor to the energy spread, and another contribution comes from longitudinal Coulomb interactions (also known as Boersch effect). The energy spread caused by the Boersch effect is predicted to be larger for the Graphene-Ni cathode compared to a typical W(310) cold field emitter (by a factor of around 20% higher for the 170 nm radius tip), but the total estimated energy spread from the combined TED distribution and Boersch effect is approximately the same (Supplementary Table 3). It is interesting to note that since both the TED distribution and Boersch effect on energy spread decrease with increasing tip radius, a significantly smaller energy spread is predicted for the 800 nm radius Graphene-Ni tip (a factor of two smaller than that of the 170 nm radius tip). These preliminary simple analytical considerations point towards new opportunities for obtaining smaller energy spreads with the Graphene-Ni cathode, which comes from its ability to produce stable field emission from relatively large cathode tip radii.

### Experimentally measured energy spread of the Graphene-Ni electron point source

**Fig. 5:**
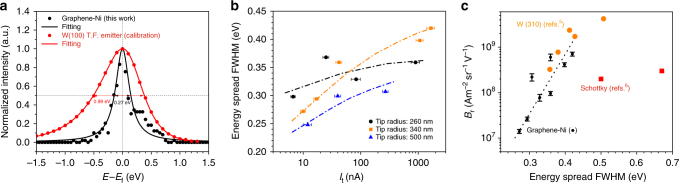
Energy spread and its dependence on emission current and reduced brightness. **a** Electron energy distribution from a Graphene-Ni tip with a radius of 340 nm emitting at a total emission current of 10 nA and an angular current of 0.1 μA sr^−1^. The pass energy and the dwell time for measurement are 10 eV and 0.1 s, respectively. The W(100) T.F. emitter was employed to calibrate to electron analyzer (Focus Electronics CSA 200). **b** Energy spread FWHM versus total emission current obtained from different tip sizes. The uncertainty in the *x*-axis (5–10%) is due to the current instability. **c** Reduced brightness versus energy spread and state-of-the-art electron sources. The uncertainty in the *y*-axis is from the determination of tip size and current instability

Figure 5a shows an experimentally measured energy spread distribution for the Graphene-Ni electron point source using a commercial high-resolution energy analyzer, shown alongside the energy spread of a W(100) thermal field (T.F.) Schottky emitter used for calibration purposes. The beam intensity in the *y*-axis is normalized to each signal’s peak height, while the energy spread on the *x*-axis is defined with respect to the Fermi Energy. The graphene-Ni tip has a radius of 340 nm, a beam voltage of 0.8 kV, while a beam voltage of 1.1 kV was used for the Schottky emitter. In both cases, the analyzer pass energy was set to 10 eV. The FWHM of 0.27 and 0.99 eV was measured for the Graphene-Ni tip and W(100) T.F. electron emitter, respectively.

Figure 5b compares data obtained for different Graphene-Ni tip radii (260 nm, 340 nm and 500 nm) (data extracted from Supplementary Fig. 8). The FWHM values increase with increasing total emission current. The lowest measured value is 0.246 eV obtained from a tip with a radius of 500 nm emitting at a total emission current of 12 nA. Plotted in Fig. 5c is the reduced brightness versus energy spread for three different emitters, shown alongside the state-of-the-art tungsten cold field emitter^5^ and Schottky emitter^6^. The error bar is calculated by error propagation due to the uncertainty in the tip radius (±30 nm) and the instability in the emission current (±5%). The overall energy spread values are lower than or comparable with state-of-the-art conventional cold field emission sources. This is due to a lower work function *ϕ* (Supplementary Note 5). As predicted by our analytical calculations, the small energy spreads expected for the Graphene-Ni cathode will be approximately off-set by the Boersch effect for the small tip sizes, and the total energy spread is comparable to conventional tungsten cold field emitters.

## Discussion

In this paper, these preliminary experimental results demonstrate that by using a few-layer graphene-coated Ni wire point cathode, it is possible to obtain stable cold field emission for electron microscopy and lithography applications in HV conditions and, additionally, use relatively large point cathode tip diameters (in the micron range). The feasibility of using such large size tips and relatively poor vacuum conditions comes from their experimentally measured ultralow work function value of 1.1 eV. The estimated reduced brightness of these new type of cold field emission sources, as well as their measured energy spread, are comparable to conventional single crystal tungsten cathode cold field emission sources. These results establish the promising prospect of using them as high brightness high-resolution electron sources for electron microscopy and lithography applications, similar in performance to conventional single crystal tungsten cathode cold field emission sources, while at the same time having better emission stability and less stringent vacuum requirements. Electron gun structures that can accommodate the Graphene-Ni electron point source need to be developed in which the source is accurately aligned with optical axis and accelerated directly after emission to avoid Coulomb interactions; this will be the subject for future studies.

## Methods

### Single Graphene-Ni tip preparation

A typical electrochemical etching process was used for preparing a sharp Ni tip having a radius of a few hundred nanometers^21^. The sharp Ni tip serves as a template and catalyst for the growth of graphene. In this work, the deposition of a few-layer graphene is achieved by using the CVD method with solid carbon source PMMA (poly(methyl methacrylate)) as feedstock, since this method avoids the use of high temperatures which may change the morphology of the sharpened tip. Supplementary Fig. 1 illustrates the schematic of the CVD setup used. As shown in the schematic, the Ni tip was placed in a ceramic holder positioned at the center of the tube furnace. An Al_2_O_3_ boat loaded with solid PMMA was placed at the inlet side of the quartz tube, just outside of the heating zone. The Ni tip was then either heated to 900 °C for tip diameters greater than 400 nm or heated up to 800–850 °C for tip diameters less than 400 nm, maintaining a 500 sccm Ar/H_2_ (95%/5%) flow. The solid PMMA source was then heated to 150 °C for 8 min after the furnace reaches the set temperature. After this, the furnace was simply opened for the precipitation/formation of few-layer graphene on the Ni tip.

### Microstructural characterization

The microscopic morphologies of the cathode were investigated by using a scanning electron microscope equipped with EDS (FEI Nova 230). The crystallinity and thickness of the graphene were examined by Raman spectroscopy (WITecCRM200 with 532 nm laser (2.33 eV excitation)) and HRTEM (JEOL 2100FEF, 200 KeV).

### Electron emission and energy spread measurements

Field emission properties of the graphene-coated point cathode were obtained in a vacuum chamber at a pressure of ~4 × 10^−8^ torr. A schematic of the experimental electron gun setup that was used to analyze the performance of the cathode is shown in Supplementary Fig. 3. The emitter tip was positioned 0.5 mm (the distance *d* was measured accurately in a SEM) away from a grounded hole anode plate having a radius of 0.5 mm, and aligned to a hole aperture (probe hole radius = 0.5 mm) that was placed in front of a Faraday Cup for beam current measurements. The solid angle of collection defined by the aperture was ~3 × 10^−3^ sr. A cylindrical sector energy analyzer (Focus Electronics CSA 200) was installed to measure the electron energy distribution. The energy analyzer was operated at constant pass energy of 10 eV and the energy scan was conducted by sweeping the retarding potential between the analyzer and the entrance focusing lens. A W(100) T.F. electron gun was employed to calibrate the electron analyzer before capturing the data.

### Data availability

The data that support the findings of this study are available from the corresponding authors upon request.

## Electronic supplementary material

Supplementary Information(PDF 1169 kb)
