# Supplementary material for: A high-brightness large-diameter graphene coated point cathode field emission electron source
Source: Nat Commun. 2018 Mar 29;9:1288. doi: 10.1038/s41467-018-03721-y (PMC5876327; doi:10.1038/s41467-018-03721-y)
Supplement: Supplementary file 1 — Supplementary Information(PDF 1169 kb) [file 41467_2018_3721_MOESM1_ESM.pdf]

SUPPLEMENTARY INFORMATION

shao *et al.*

A high-brightness large-diameter graphene coated point cathode field emission electron source

This PDF File includes:

Supplementary Figure 1-8

Supplementary Note 1-5

Supplementary Table 1-3

Supplemetary Reference

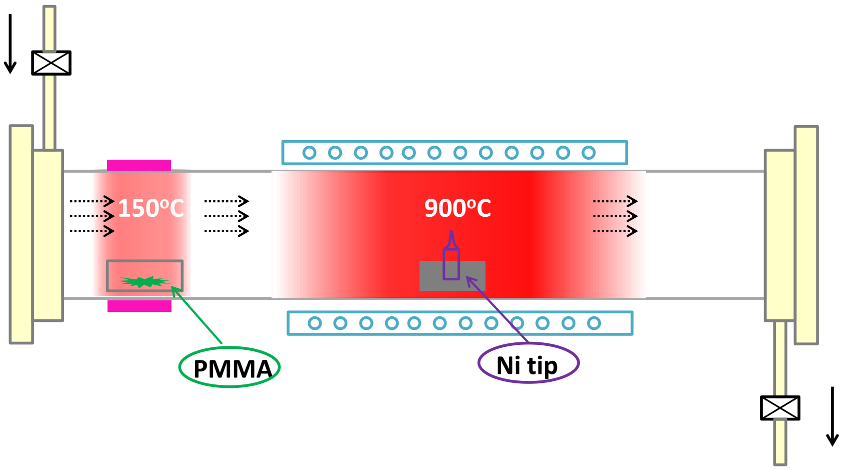

Supplementary Figure 1 | Chemical vapor deposition growth of graphene. The schematic illustration of the CVD set up.

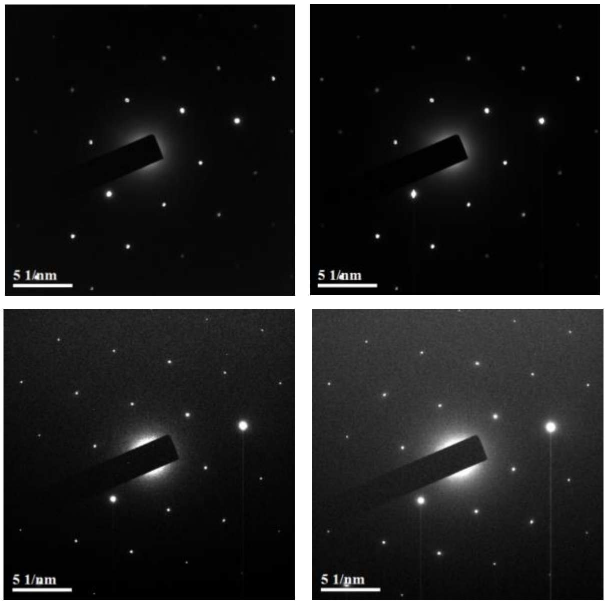

Supplementary Figure 2 | Selected area electron diffraction (SAED) pattern. SAED acquired on different regions of the graphene surface.

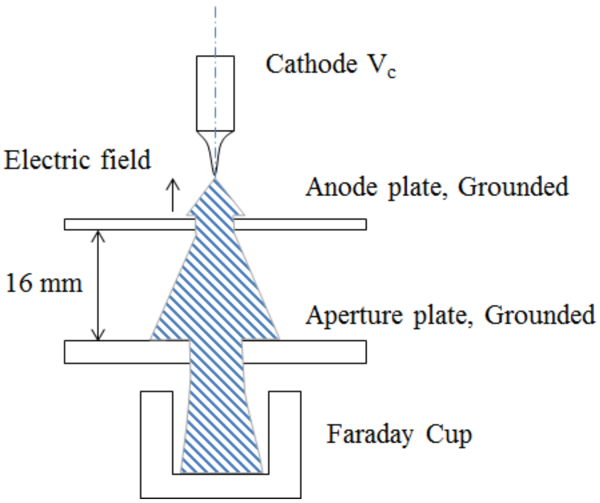

Supplementary Figure 3 | Electron gun test set up. Schematic diagram of the experimental electron gun that was used to measure I'.

Supplementary Note 1 | Work function measurements

According to the simulations from Lorentz-2EM boundary element software[1], one example of equipotential plot in equal voltage intervals is shown in Supplementary Fig. 4(a). The cathode is biased at -1200 V relative to the grounded anode 1 which is placed at 0.5 mm away. The resulting local field strength F was found to be 0.68 V nm^{-1} on the tip as shown in Supplementary Fig. 4(b). In this diagram, the field enhancement factor β is defined as the ratio of the local electric field strength at the apex F over the applied field E, which is equal to the cathode voltage, U, divided by the cathode-tip to anode distance, d, i.e.

$$\beta = \frac{F}{E} = \frac{Fd}{U} \qquad (1)$$

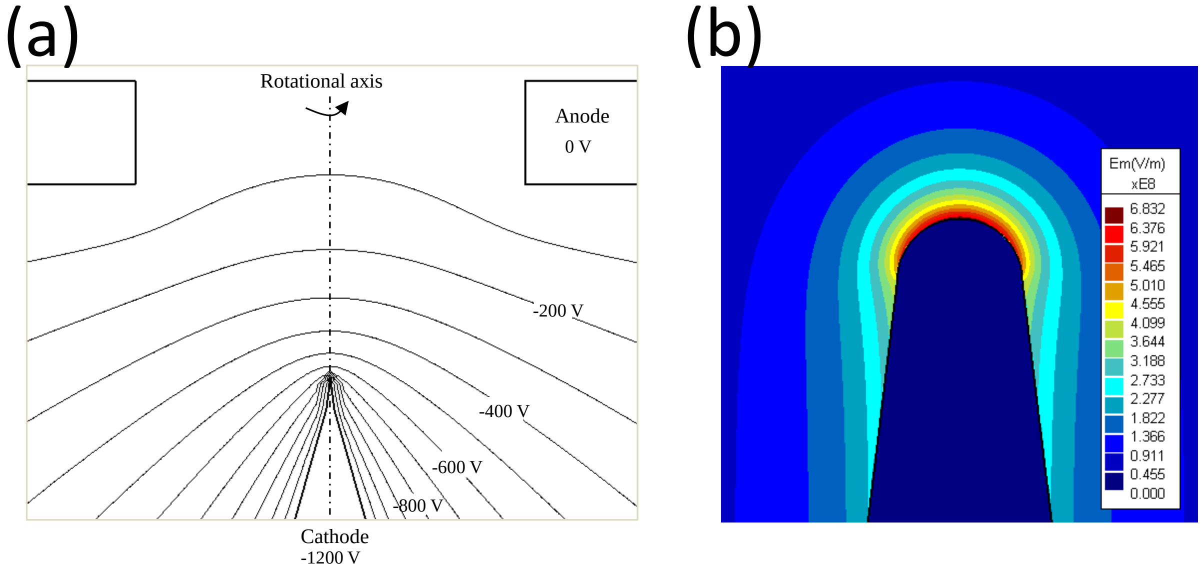

Supplementary Figure 4 | Local tip field strength. (**a**) An example of equipotential plot in equal voltage intervals. The cathode is biased at -1200 V relative to the anode which is placed at 0.5 mm away. (**b**) A contour plot of electric field strength on the emitter tip of radius 500 nm. The applied voltage is -1200 V and the resulting local field strength is 0.68 V nm^{-1} on the tip.

The Fowler-Nordheim (F-N) law is normally used to describe the field emission behavior from metallic surface, and is given by[2]:

$$I = A\frac{1.5\times10^{-6}}{\phi}(\frac{U}{d})^2\beta^2\exp(-\frac{6.44\times10^9\phi^{1.5}d}{\beta U}) \qquad (2)$$

This Supplementary Eq. (2) can be re-written as:

$$\ln\left(\frac{I}{U^2}\right) = \ln\left(\frac{1.5\times10^{-6}\beta^2 A}{d^2\phi}\right) - \frac{6.44\times10^9\phi^{1.5}d}{\beta}\left(\frac{1}{U}\right) \qquad (3)$$

A plot of $\ln(I/U^2)$ against $1/U$ will have a slope of $m = -(6.44 \times 10^9\phi^{1.5}d/\beta)$. This slope depends on ϕ, d, and β. Since the value of d is fixed in the experimental setup, and m is obtained from the slope of the F-N plot, the work function value ϕ can be estimated if the field enhancement factor β is known. The field enhancement factor β can be extracted from performing direct ray tracing of electron trajectory paths by simulation as shown above (Supplementary Eq. 1), and the work function can be calculated by:

$$\phi = \sqrt[1.5]{\frac{-m\beta}{6.44\times10^9 d}} \qquad (4)$$

However, it is important to first validate the accuracy of this approach by using it to experimentally measure the work function of the bare Ni tip, before it is coated with graphene. The work function for the bare Ni tip is expected to agree with previous reported values for bulk Ni[3]. The work function measurements for bare Ni tip using Supplementary Eq. 4 were found to be 5.80 eV (compared to 5.47 eV for bulk Ni), showing an accuracy of 94%. Once this has been established, the work function for the Ni coated graphene can be found by using two F-N plots, one for the bare Ni tip, and one for the graphene coated Ni tip, and then take the ratio of their F-N slopes, eliminating d and β, and calibrate the graphene coated work function value relative to that measured for the bare Ni tip (verified by comparison to previous reported values for bulk Ni).

This procedure assumes that the addition of graphene does not change the tip geometry (confirmed by SEM imaging). From the ratio of the two F-N slopes, the effective work function is calculated from:

$$\phi_{\mathrm{Graphene+Ni}} = \phi_{\mathrm{Ni}} \sqrt[1.5]{\left(\frac{m_{\mathrm{Graphene+Ni}}}{m_{\mathrm{Ni}}}\right)} \qquad (5)$$

Where $\phi_{\mathrm{Graphene+Ni}}$ and ϕ_{Ni} are the work functions of graphene coated pointed cathode and bare Ni cathode, $m_{\mathrm{Graphene+Ni}}$ and m_{Ni} are the slopes of the F-N plot for graphene coated point cathode and bare Ni cathode, respectively. The local electric field strength F and β were obtained by numerically solving for the electric potential distribution using the Lorentz-2EM software.

Supplementary Note 2 | Determination of the source reduced brightness B_r.

Trajectory ray-tracing simulation is used to compute the exact value of angular magnification $m_\alpha = \alpha/\theta$, where α is the final extraction angle and θ is the initial emission angle. The cathode emission area S_p (Supplementary Fig. 5) is given by:

$$S_p = 2\pi \times r_{tip}^{\;2}(1 - \cos\theta) \qquad (6)$$

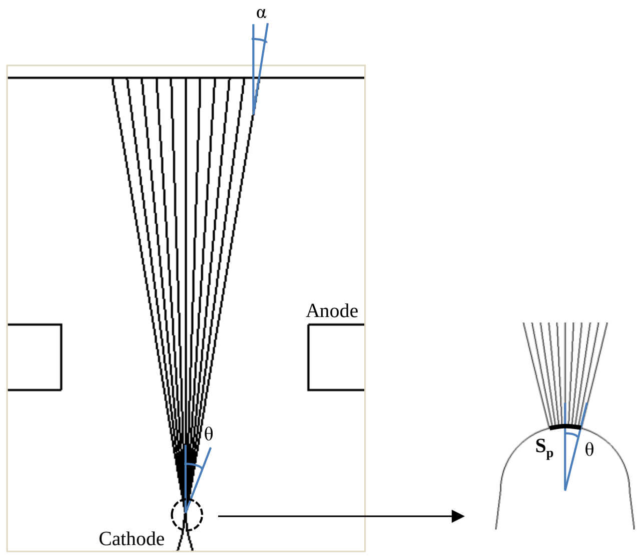

Supplementary Figure 5 | Determination of the angular magnification. Angular magnification $m_\alpha = \alpha/\theta$, where α is the final extraction angle and θ is the initial emission angle.

By using a Faraday Cup with a small acceptance aperture (restricting the semi-angle entry to 30 mrad), emission current is collected only from a small cathode emission area S_p. For tip radii in the range of 130-800 nm, S_p was estimated to be in the range of 130-3965 nm^2 (calculated from Supplementary Eq. 6). These S_p values are much smaller than the individual domain sizes bounded by wrinkles in the graphene coating (typically observed for areas greater than 1 µm×1 µm), eliminating the possibility that emission from wrinkles in the graphene coating contributed to the current collected by the Faraday Cup.

The source reduced brightness is defined by the following relationship[4]:

$$B_{\mathrm{r}} = \frac{4I'}{\pi d_{\mathrm{v}}^2 V_{\mathrm{ext}}} \quad (7)$$

where I' is the angular current density, d_{v} is the virtual source size, and V_{ext} is the extraction voltage. The virtual source size can be calculated using the derived formula[4]:

$$d_{\mathrm{v}} = 1.67 \frac{r_{\mathrm{tip}}}{m_{\alpha}} \sqrt{\frac{<E_{\mathrm{t}}>}{eV_{\mathrm{ext}}}} \quad (8)$$

In this formula, r_{tip} is the tip radius and $< E_{\mathrm{t}} >$ is defined as $< E_{\mathrm{t}} > = e\hbar F/\sqrt{(8m\phi)}$, with F the local electric field strength, ϕ the work function, and $\hbar$ the reduced Planck constant. Using Supplementary Eqs. 7 and 8 gives the following formula for source reduced brightness for cold field emitters[4]:

$$B_{\mathrm{r}} = 1.44 \frac{eI'}{\pi<E_{\mathrm{t}}>} \left(\frac{m_{\alpha}}{a}\right)^2 \quad (9)$$

Tip No.	r_{tip} (nm)	F (Vnm^{-1})	I' (μAsr^{-1})	m_{α}	θ(rad)	d_{v}(nm)	B_{r} (Am^{-2}sr^{-1}V^{-1})
1	130	1.178	12.1	0.613	0.049	3.54	1.12×10^{9}
2	170	0.958	40.7	0.559	0.054	4.55	2.51×10^{9}
3	270	1.067	12.0	0.654	0.046	6.55	3.24×10^{8}
4	290	1.109	23.6	0.656	0.046	6.85	5.36×10^{8}
5	400	0.781	45.5	0.629	0.048	7.95	7.09×10^{8}
6	480	0.527	7.8	0.623	0.049	10.08	1.23×10^{8}
7	520	0.695	6.7	0.643	0.047	9.18	7.25×10^{7}
8	700	0.788	16	0.682	0.044	10.94	9.48×10^{7}
9	800	0.564	11.4	0.682	0.044	11.79	7.23×10^{7}

Supplementary Table 1 | Data used for the calculation of the reduced brightness. The data of 9 cathode-tips used for the calculation of the reduced brightness that are shown in Supplementary Fig. 4a. θ denotes the initial emission angle. Others are the same as described in the article.

Supplementary Note 3 | Coulomb interactions.

It is well known that a high brightness electron source may encounter adverse statistical Coulomb effects. These effects manifest as energy broadening or more commonly known as Boersch effect (due to longitudinal interactions) and radial broadening (due to lateral interactions), and thus degrades the quality of an electron source. The former adds to the intrinsic source energy spread while the latter causes an additional enlargement of the virtual source size which effectively reduces the source brightness. The statistical Coulomb effects can be estimated in an approximate way by Monte Carlo numerical simulations, and also by simple analytical approximations[5,6].

Boersch effect. According to the Knauer's model[5] of a spherical electric field around an emitter of tip radius r_{tip}, the energy broadening (in eV) due to Coulomb interactions is given by the formula:

$$\Delta E_{\text{Boersch}} = 15.9\frac{(I')^{2/3}}{r_{\text{tip}}^{1/3}V_{\text{ext}}^{1/3}} \quad (10)$$

where I' is the angular current density and V_{ext} is the extraction voltage.

Radial broadening and brightness correction. The slice method[7] is used to calculate the Coulomb interactions, in which the region to be calculated is divided into small segments over which the voltage and beam size is assumed to remain constant. The trajectory displacement is calculated by applying this slice method to the analytical approximation in the gun region.

$$d_{\text{blur}} = \int \frac{l(z)}{M(z)}\phi_J(r(z), V(z), I)\, dz$$

Where $l(z) = r(z)/\alpha(z)$, and $M(z)$ is the magnification ($= (\frac{\alpha_{\text{ref}}}{\alpha(z)})\sqrt{(\frac{V_{\text{ref}}}{V(z)})}$).

The slice method requires the angular displacement per meter, given by:

$$\phi_J = \left[\frac{T_1 D_\lambda^{18/7} D_r^6 I^{18/7} r^{6/7} V^{-15/7}}{T_4 + T_2^{1/7} D_r^6 D_\lambda^2 I^2 r^2 V^{-1}}\right]^{7/6} \quad (11)$$

Where $D_\lambda = m^{\frac{1}{2}}/(\pi 2^{\frac{7}{2}} \epsilon_0 e^{\frac{1}{2}})$ and $D_r = (\frac{2\epsilon_0 \pi}{e})^{1/3}$. The constant are $T_1 = 4.618 \times 10^{-2}$, $T_2 = 2.041 \times 10^5$, and $T_4 = 6.25 \times 10^{-2}$. $V(z)$ and $r(z)$ are determined using the ray tracing simulations.

The integration is done with the Simpson's 1/3 rule applied to unequal intervals. Radial broadening has the effect of increasing the intrinsic virtual source size d_v, which in turn will lower the brightness estimate by a correction factor K given by:

$$K = \frac{d_v^2}{d_v^2 + d_{blur}^2} \quad (12)$$

The values of d_{blur} and K are given in Supplementary Table 2 for a few selected Graphene-Ni tips. Also included in the table is the corrected brightness KB_r. The results predict that the brightness will only be lowered by Coulomb interaction effects significantly for the smaller Graphene-Ni cathode-tip radius (reduction by around 40% for the 170 nm tip radius), and is not expected to significantly lower the brightness estimates for the larger Graphene-Ni cathode tip radii.

r_{tip} (nm)	d_v (nm)	d_{blur} (nm)	K	B_r ($Am^{-2}sr^{-1}V^{-1}$)	KB_r ($Am^{-2}sr^{-1}V^{-1}$)
170	4.55	3.86	0.581	2.51×10^9	1.46×10^9
400	8.27	2.75	0.900	7.09×10^8	6.38×10^8
800	11.79	0.79	0.996	7.23×10^7	7.20×10^7

Supplementary Table 2 | Radial broadening predictions for the Graphene-Ni tips. Radial broadening effect has been calculated on three typical Graphene-Ni tips.

Supplementary Note 4 | Measurements of the emission repeatability.

Eighteen cycles of I'-V curves were obtained from a cathode of tip radius of 700 nm (Supplementary Fig. 6a), where the angular current density was kept below 2 μA sr^{-1}. Beyond the initial eight runs, a shift of the $I' - V$ curve to the right was observed after which it remained stable with no further shift. After leaving the tip in the HV chamber for 25 days without emission, another round of twenty cycles of $I' - V$ curves were measured, as shown in Supplementary Fig. 6b. The $I' - V$ characteristics largely look similar in both rounds of testing. The shift of the $I' - V$ curve to the right after some initial cycles may be attributed to the expelling of the adsorbate molecules over time and can be considered as a part of a "preconditioning" process. These results confirm that the graphene coated point cathode has highly repeatable field emission characteristics.

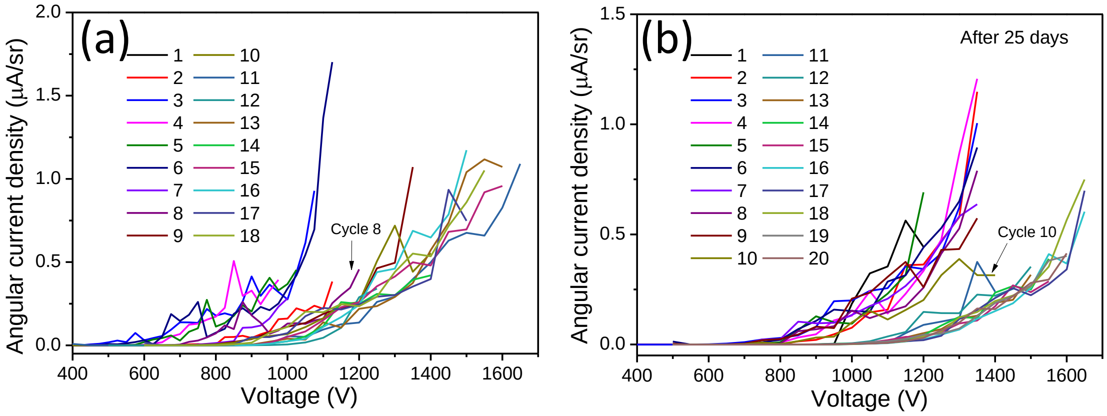

Supplementary Figure 6 | The emission repeatability. (a) Cyclic measurements of field emission of a 700 nm radius tip graphene coated cathode. (b) Cyclic measurements taken from the same tip after 25 days.

Supplementary Note 5 | Energy spread.

Analytical calculations. An analytical expression for the total energy distribution (TED) of electron emission in the thermal field regime was first derived by Young[8] based on the free-electron model as:

$$P(E) = \frac{J_{\mathrm{FN}}}{d}\left[\frac{\exp\left(\frac{E-E_{\mathrm{f}}}{d}\right)}{1+\exp\left(\frac{E-E_{\mathrm{f}}}{kT}\right)}\right] \qquad (13)$$

where kT = 0.155 eV at room temperature, J_{FN} is the well-known Fowler-Nordheim emission current density and d is the tunneling parameter (in eV) given by:

$$d = 9.76 \times 10^{-11}\frac{F}{\phi^{1/2}t(y)} \qquad (14)$$

where F and ϕ are the electric field strength (in V m^{-1}) and work function (in eV) respectively. The variable $t(y)$ is a slowly-varying function of $y = 3.79\times10^{-5} F^{1/2}/\phi$ and can be approximated by the formula $t(y) = 1 + 0.1107\, y^{1.33}$. The analytical formula is valid[9] provided $kT/d < 0.7$ and $y < 1$.

Supplementary Figure 7 shows the room-temperature TED for a typical W(310) cold field emitter[10] at ϕ = 4.32 eV, F = 4.34 V nm^{-1} as well as for a graphene-coated nickel tip at ϕ = 1.10 eV, F = 0.781 V nm^{-1}. From these plots, the intrinsic full-width at half-maximum (FWHM) energy spreads are calculated to be 0.23 and 0.14 eV respectively. The smaller predicted intrinsic FWHM of the Graphene-Ni cathode compared to the typical W(310) cold field emitter (by 60%), comes mainly from its smaller field strength requirement.

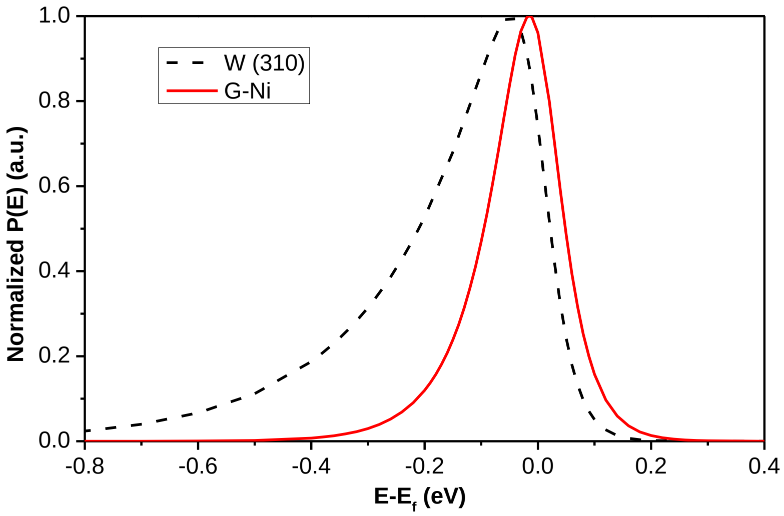

Supplementary Figure 7 | Analytical calculations of the energy spread. Room-temperature total energy distributions for a typical W(310) cold field emitter (ϕ = 4.32 eV, F = 4.34 V nm^{-1}) and a Graphene-Ni emitter (ϕ = 1.10 eV, F = 0.781 V nm^{-1}). The FWHM energy spreads are 0.23 and 0.14 eV respectively.

The intrinsic TED of electron emission is only one contributor to the energy spread, and another contribution comes from Coulomb interactions. Unlike the situation for TED, lower electric fields (lower extraction voltage), enlarge the energy spread caused by the Boersch effect, according to Knauer's model (Supplementary Eq. 10).

As a first approximation, the total source energy spread can be calculated from quadratic addition (root-mean-square sum) of the Boersch effect and the intrinsic energy spread calculated from the TED:

$$\Delta E_{total} = (\Delta E_{intrinsic}^2 + \Delta E_{Boersch}^2)^{1/2} \qquad (15)$$

Values of $\Delta E_{\mathrm{intrinsic}}$, $\Delta E_{\mathrm{Boersch}}$, and $\Delta E_{\mathrm{total}}$ are given in Supplementary Table 3 for a typical W(310) tip[10] and a few selected Graphene-Ni tips.

Source	r_{tip} (nm)	V_{ext} (V)	I' (μAsr^{-1})	d_{v} (nm)	B_{r} (Am^{-2}sr^{-1}V^{-1})	$\Delta E_{\mathrm{intrinsic}}$ (eV)	$\Delta E_{\mathrm{Boersch}}$ (eV)	$\Delta E_{\mathrm{total}}$ (eV)
W(310)	160	4255	62	2.98	2.09×10^{9}	0.232	0.283	0.366
Graphene-Ni	170	975	40.7	4.55	2.51×10^{9}	0.144	0.342	0.371
Graphene-Ni	400	1300	45.5	8.27	7.09×10^{8}	0.140	0.252	0.288
Graphene-Ni	800	1450	11.4	11.79	7.23×10^{7}	0.136	0.077	0.156

Supplementary Table 3 | Energy spread predictions. The total energy spread comes from the intrinsic TED of electron emission and Coulomb interactions. Both values have been semi-analytically calculated.

The results in Supplementary Table 3 show that the energy spread caused by the Boersch effect is predicted to be larger for the Graphene-Ni cathode compared to a typical W(310) cold field emitter (by a factor of around 20% higher for the 170 nm radius tip), but the total estimated energy spread from the combined TED distribution and Boersch effect is approximately the same. These considerations indicate that for the smaller tip sizes (around 170 nm radius), the smaller energy spreads expected for the Graphene-Ni cathode compared to conventional tungsten cold field emitters (of comparable tip size) based upon the TED distribution, will be approximately off-set by the Boersch effect, and the total energy spread for the two emitters is therefore expected to be comparable.

It is interesting to note that since both the TED distribution and Boersch effect on energy spread decrease with increasing tip radius, a significantly smaller energy spread is predicted for the 800 nm radius Graphene-Ni tip (a factor of two small than that of the 170 nm radius tip). This would ordinarily not be possible for conventional large field emitters (tip-diameters over one micron), such as the Schottky emitter, since the Schottky field emitter only functions by heating

the tip up to 1800 K, enlarging the energy spread by thermal effects to around 0.5 eV. These preliminary simple analytical considerations point towards new opportunities for obtaining smaller energy spreads with the Graphene-Ni cathode, which comes from its ability to produce stable field emission from relatively large cathode-tip radii.

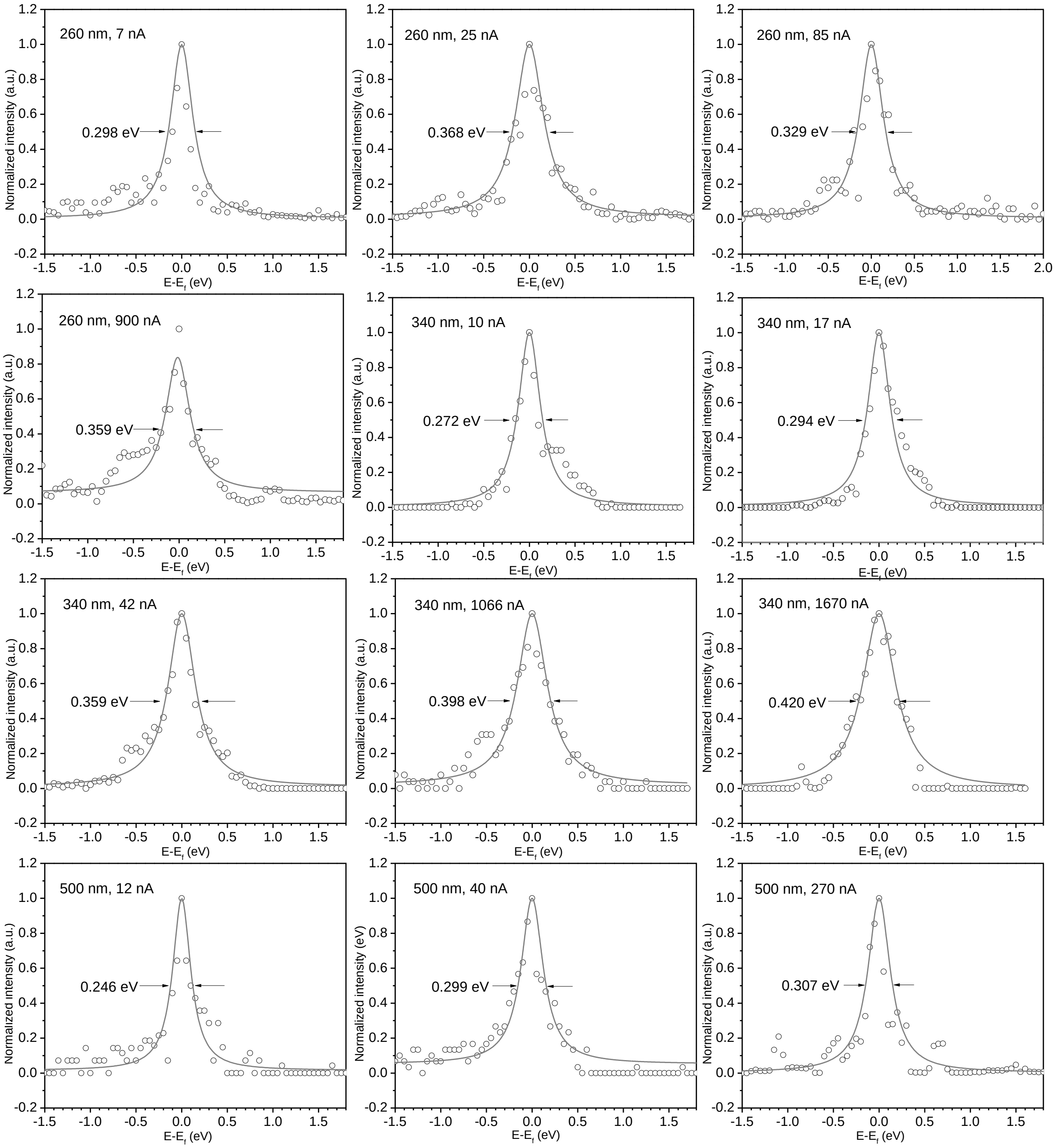

Supplementary Figure 8 | Experimentally measured energy spread of the Graphene-Ni electron point source. Detailed electron energy distribution spectra of those used in Supplementary Fig. 5b.

References:

1 Lorentz-2EM, v. 9.3. (Integrated Engineering Software Inc., Canada, 2015).

2 Fowler, R. H. & Nordheim, L. in *Proceedings of the Royal Society of London A: Mathematical, Physical and Engineering Sciences.* 173-181 (The Royal Society).

3 Giovannetti, G. *et al.* Doping graphene with metal contacts. *Physical Review Letters* **101**, 026803 (2008).

4 Bronsgeest, M., Barth, J., Swanson, L. & Kruit, P. Probe current, probe size, and the practical brightness for probe forming systems. *Journal of Vacuum Science & Technology B: Microelectronics and Nanometer Structures Processing, Measurement, and Phenomena* **26**, 949-955 (2008).

5 Knauer, W. ENERGY BROADENING IN FIELD EMITTED ELECTRON AND ION-BEAMS. *Optik* **59**, 335-354 (1981).

6 Kruit, P. & Jansen, G. H. Space charge and statistical Coulomb effects. *Handbook of Charged Particle Optics* **2** (1997).

7 Cook, B., Verduin, T., Hagen, C. & Kruit, P. Brightness limitations of cold field emitters caused by Coulomb interactions. *Journal of Vacuum Science & Technology B, Nanotechnology and Microelectronics: Materials, Processing, Measurement, and Phenomena* **28**, C6C74-C76C79 (2010).

8 Young, R. D. Theoretical total-energy distribution of field-emitted electrons. *Physical Review* **113**, 110 (1959).

9 Bahm, A., Schwind, G. & Swanson, L. Range of validity of field emission equations. *Journal of Vacuum Science & Technology B: Microelectronics and Nanometer Structures Processing, Measurement, and Phenomena* **26**, 2080-2084 (2008).

10 Swanson, L. & Schwind, G. A review of the cold-field electron cathode. *Advances in imaging and electron physics* **159**, 63-100 (2009).
